# Comment on “Mortality Due to Aortic Dissection in Adults With Primary Hypertension: A Nationwide Analysis Over Two Decades”

**DOI:** 10.1002/clc.70320

**Published:** 2026-04-30

**Authors:** Muhammad Faizan Khan, Muhammad Imran

**Affiliations:** ^1^ Nowshera Medical College Nowshera Pakistan; ^2^ Khyber Medical University Peshawar Pakistan

Dear Editor,

We read with great interest the article by Ahmad et al. [1] which is a descriptive study of the US population over two decades. The study presents a descriptive analysis of the US mortality trends and demographic disparities in death due to aortic stenosis coexisting hypertension. The study provides useful population‐level analysis and observations, however, some of the methodological considerations substantially limits interpretation of the findings and warrant clarification.

One of the major limitations is that the outcomes are framed as mortality among adults with primary hypertension where all reported rates are calculated using the general US population as the denominator rather than the population of individuals with hypertension. Consequently, these estimates reflect population‐level mortality in which hypertension was recorded on death certificates, not mortality risk within hypertensive patients. This distinction is not explicitly emphasized and introduces the risk of ecological misinterpretation [2].

Compounding this issue, the dataset contains no individual‐level clinical information. Despite this, the Discussion suggests that improved hypertension control may explain observed mortality trends. However, in the absence of measured exposure or treatment variables, such interpretations remain speculative and exceed what population‐based mortality data can support, particularly given evidence of declining hypertension control in recent years [3]. Furthermore, Type A and Type B aortic dissections were analyzed together despite substantial differences in pathophysiology and prognosis, potentially obscuring subtype‐specific trends and limiting clinical interpretability [4].

Although the analysis offers important population‐level observations, methodological constraints limit inference regarding mortality risk among individuals with hypertension. In addition, individual‐level data were unavailable, and subtype‐specific pathophysiology and prognosis could not be distinguished, which limits clinical interpretability. Future investigations would be strengthened by incorporating hypertensive population denominators, individual‐level clinical data, and stratification by aortic dissection subtype. Linkage of mortality records with clinical registries or claims databases could help disentangle incidence from case fatality and allow more precise assessment of subgroup risk.

## Funding

The authors have nothing to report.

## Ethics Statement

The authors have nothing to report.

## Consent

The authors have nothing to report.

## Conflicts of Interest

The authors declare no conflicts of interest.

## Data Availability

The authors have nothing to report.
